# A primer for ZooMS applications in archaeology

**DOI:** 10.1073/pnas.2109323119

**Published:** 2022-05-10

**Authors:** Kristine Korzow Richter, Maria C. Codlin, Melina Seabrook, Christina Warinner

**Affiliations:** ^a^Department of Anthropology, Harvard University, Cambridge, MA 02318;; ^b^Department of Anthropology, Boston University, Boston, MA 02215;; ^c^Department of Archaeogenetics, Max Planck Institute for Evolutionary Anthropology, Leipzig 04103, Germany

**Keywords:** mass spectrometry, MALDI-TOF, peptide mass fingerprinting, zooarchaeology

## Abstract

Collagen peptide mass fingerprinting by matrix-assisted laser desorption/ionization time-of-flight (MALDI-TOF) mass spectrometry, also known as zooarchaeology by mass spectrometry (ZooMS), is a rapidly growing analytical technique in the fields of archaeology, ecology, and cultural heritage. Minimally destructive and cost effective, ZooMS enables rapid taxonomic identification of large bone assemblages, cultural heritage objects, and other organic materials of animal origin. As its importance grows as both a research and a conservation tool, it is critical to ensure that its expanding body of users understands its fundamental principles, strengths, and limitations. Here, we outline the basic functionality of ZooMS and provide guidance on interpreting collagen spectra from archaeological bones. We further examine the growing potential of applying ZooMS to nonmammalian assemblages, discuss available options for minimally and nondestructive analyses, and explore the potential for peptide mass fingerprinting to be expanded to noncollagenous proteins. We describe the current limitations of the method regarding accessibility, and we propose solutions for the future. Finally, we review the explosive growth of ZooMS over the past decade and highlight the remarkably diverse applications for which the technique is suited.

Zooarchaeology by mass spectrometry (ZooMS) is a powerful application of collagen peptide mass fingerprinting (PMF) first developed just over a decade ago (1). Based on the measurement of tryptic collagen peptides using a matrix-assisted laser desorption/ionization time-of-flight (MALDI-TOF) mass spectrometer, it leverages the high abundance and long-term preservation of collagen in bone and other animal tissues with the analytical power of mass spectrometry (MS) in order to provide robust taxonomic identifications using minimally destructive methods. Since 2009, ZooMS has been used for diverse applications in archaeology and paleontology, ecology and conservation, and cultural heritage. The key features of ZooMS that have led to its rapid expansion are its low sample input requirements and its relatively low analytical cost per sample compared with other biomolecular identification methods. This allows for large-scale taxonomic investigations that can augment morphological analyses of faunal assemblages as well as provide taxonomic clarity for animal remains or products lacking diagnostic features, as is common for worked bone artifacts and cultural heritage objects (2).

## ZooMS One Decade in

Over the past decade, ZooMS has been widely used to provide identifications of collagenous materials, including bone (1), ivory (3), antler (4), parchment and vellum (5), leather (6), and other soft tissues (7). Within the context of archaeology, the application of ZooMS to faunal assemblages has allowed a wide range of topics to be explored, including domestic herd management, choices relating to secondary product use, exploitation of wild species, and the appearance of commensal species (5,8–12) (*SI Appendix*). Improvements in scalability, automation, and high-throughput processing mean ZooMS can be used as a screening tool in order to identify species of interest among otherwise unidentifiable fragmentary remains. This approach has been highly successful in identifying a handful of hominin remains from nearly 10,000 bone fragments at Denisova cave (13–15), human remains at other Late Pleistocene and Early Holocene sites (16,17), and extinct megafauna (18).

Better taxonomic resolution of assemblages has also allowed for improved ecological reconstructions. ZooMS is best applied in situations when morphologically similar species inhabit different ecological niches. This is applicable in the reconstruction of terrestrial ecosystems (19,20), but ZooMS is even more powerful when applied to the reconstruction of aquatic ecosystems due to the larger number of possible species usually present and the reduced ability to achieve desired taxonomic resolution using conventional techniques (21,22). Although the integration of findings from ZooMS data into current conservation practices remains limited, recent ZooMS successes in identifying ivory (3) and distinguishing wild African bovids (11,23) show great promise for providing low-cost solutions for identifying the trade of illicit animal products, such as ivory objects and bushmeat.

Finally, because ZooMS uses a very low starting amount of material and can be performed with minimally destructive and noninvasive protocols, it is an ideal method for the identification of worked bone tools and other composite artifacts from both archaeological and cultural heritage contexts. These include worked bone and antler (bone points, arrowheads, daggers, rings, combs), leather, composite artifacts, parchment, works of art, and gelatin-coated photographs (5–7,24–29). In addition, it has been used to help identify remains in museum collections in cases where the associated metadata have been lost (30).

The first decade of ZooMS has showcased its wide-ranging, continued applicability (*SI Appendix*, Fig. S1). Although tandem mass spectrometry and ancient DNA approaches provide higher taxonomic resolution, PMF-based methods are more cost effective, allow for greater sample throughput, and provide sufficient taxonomic resolution for many archaeological and cultural heritage questions, making them more accessible for many researchers. The past decade has also highlighted the need for further ZooMS development, particularly with respect to standardizing data reporting, centralizing marker databases, using consistent nomenclature, and developing automated tools for data analysis. Addressing these limitations will allow ZooMS to grow from the purview of a small number of research laboratories into a robust, widely used method. Here, we review the state of the field in ZooMS research. After detailing what collagen is and why it is important in archaeological and cultural heritage research, we describe the major technological advancements that led to the development of ZooMS and the growth of its subsequent applications. We review how the method has changed and expanded over the past decade, and we outline current limitations in the field. Finally, we discuss the outlook of ZooMS research over the next decade.

## Collagen: What It Is, and Why It Matters

**Fig. 1.**
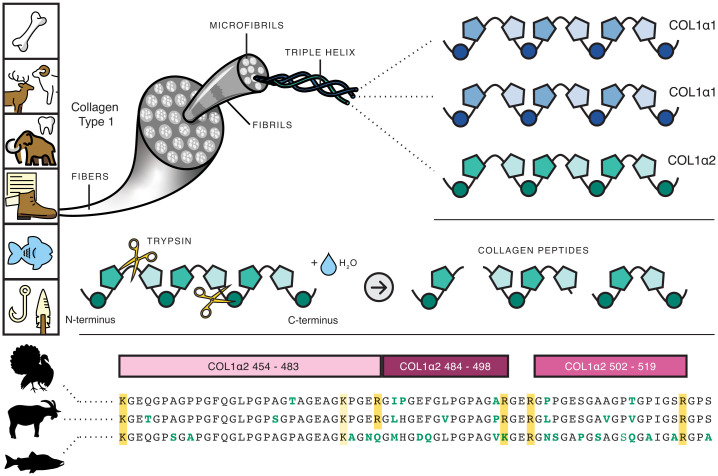
Overview of collagen structure and archaeological sources. Collagen can be retrieved from a wide range of animal tissues. In most animals, the COL1 triple helix is composed of two ɑ1-chains and one ɑ2-chain. Five triple helices are bundled into a microfibril. Bundles of microfibrils form a fibril, and bundles of fibrils form fibers. During the initial stages of ZooMS, this structure is denatured, allowing the enzyme trypsin to cut the protein into peptides. Peptides differ in sequence and mass across taxa, as shown for turkey (*M. gallopavo*), goat (*C. hircus*), and coho salmon (*O. kisutch*). Icons are fromhttps://openmoji.organdhttps://smart.servier.com. Adapted from ref.^42^.

Collagens are an abundant class of structural proteins essential for life in animals, from sponges to humans (31). There are nearly 30 different collagen proteins, of which type I collagen (COL1) is among the most ubiquitous and abundant (32), comprising 80% of the bone proteome (33). COL1 is the major component of animal connective tissues. It is highly abundant in skin, tendon, ligament, and fish scales (33,34), and it is also found in bird and reptile eggshells (35,36), invertebrate shells (37), and a wide range of other animal-derived tissues. Common archaeological remains and cultural heritage objects that contain collagen include bone, cartilage, antler, horn cores (but not horn itself), tooth dentine and cementum, ivory, parchment, leather, fish scales, and composite tools or artwork containing sinews, animal glues, or animal binders (Fig. 1).

### COL1 Fibril Formation and Structure.

At the molecular level, COL1 consists of a triple helix made up of three polypeptide α-chains (COL1ɑ) (38). In tetrapods, the triple helix is heterotrimeric, composed of two identical COL1ɑ1 chains and one COL1ɑ2 chain (Fig. 1). In teleost fish, it is made up of three different chains (COL1ɑ1, COL1ɑ2, COL1ɑ3) (39), while a small number of species, such as the unicellular hydra, have homotrimeric COL1 composed of three COL1ɑ1 chains. The amino acid sequence of COL1 is highly structurally and functionally constrained (31). Each chain consists of a repeating motif of G-X-Y (Fig. 1) with glycine (G), the smallest amino acid, fitting into the central core of the rotating triple helix. The remaining X and Y amino acid positions are disproportionately made up of proline and hydroxyproline, respectively, the latter being a posttranslational modification (PTM) of proline rarely found outside of collagens. Hydroxyprolines stabilize the triple helix through hydrogen bonding (40,41) and can be always present (fixed modification) or variably present (variable modification) at a given amino acid position. Amino acids with bulky functional groups are almost entirely absent from COL1 because they disrupt or prevent the formation of the triple helix.

During COL1 formation, proto-COL1ɑ chains are produced containing a signal peptide (∼20 amino acids) and N-terminal and C-terminal propeptides (∼150 to 300 amino acids each) that flank the mature ɑ-chain (∼1000 amino acids). The signal peptide aids in trafficking the ɑ-chain to the endoplasmic reticulum, where the propeptides initiate triple-helix formation. The signal peptide and propeptides are removed prior to fibrillogenesis, and thus are not of interest for ZooMS (38,42). The mature COL1 protein consists of a highly conserved triple-helical region flanked at each end by short, highly variable telopeptides (∼9 to 30 amino acids). After cellular secretion, the collagen triple helices aggregate into groups of approximately five to form microfibrils (43). Groups of microfibrils aggregate to form fibrils, which then bundle together to form collagen fibers (Fig. 1). In bone, collagen fibers serve as the template for biomineralization (44). The tight packing and bundling of collagen are essential to its function as a structural protein and contribute to collagen’s long-term persistence and preservation in the archaeological record (45,46).

### Collagen Degradation and Recovery in Various Archaeological Tissues.

Collagen preservation has been extensively studied, in part because of the importance of bone collagen for stable isotope analysis and radiocarbon dating (45,47). Studies of paleontological and archaeological collagen have revealed that well-preserved, high molecular weight collagen (>30 kDa) in excess of 30% of the original protein length can still be found even within extremely old bones (>1 Mya) (45,48–50). Despite its abundance and robusticity, however, collagen is nevertheless still susceptible to taphonomy and diagenesis. A wide range of soil microbes and fungi are capable of producing collagenases that rapidly degrade unmineralized collagen, and even mineralized collagen undergoes spontaneous chemical hydrolysis of peptide bonds (51). Decomposing soft tissue contributes to bone demineralization by exposing collagen to microbial attack, and the secretion of organic acids from microbial growth further demineralizes skeletal material (46,52). Individual amino acids can undergo diagenetic alterations, such as deamidation, glycation, oxidation, and cross-linking, to produce diagenetiforms, which disrupt the triple-helical structure and make the collagen backbone more susceptible to hydrolysis (45,53,54). Over time, the integrity of the collagen protein within archaeological remains declines, and collagen degradation products begin to leach out.

For archaeological remains, collagen preservation is typically estimated by measuring the dry weight percentage (%wt) of collagen or by determining the percentage of N (%N) or the atomic C:N of a given collagenous material (49,55,56). Depending on the species and type of bone, fresh bone contains 20 to 35% organic matrix, of which ∼90% is collagen (57,58), giving bone a %N of ∼3.5 to 4.5% (55), and, for humans, a C:N of 3.2 (59). As a general rule, a minimum of 1% collagen, 0.5% N, and C:N values between 2.9 and 3.6 are widely used as a minimum standard for collagen preservation in stable isotope and radiocarbon dating studies (55,59). Even less material is required for ZooMS, which has been shown to reliably yield identifiable collagen spectra from bones with as little as 0.26% N (60). As such, ZooMS can generally be applied to a wider range of archaeological remains, including from challenging environments and deep time that would otherwise be ill suited for other methods (61).

## PMF: The Mass Spectrometry Revolution for Paleoproteomics and Cultural Heritage

### Peptide Mass Fingerprint Basics.

PMF is a technique, developed in the 1990s (62), to identify proteins by the masses of the peptides produced following enzymatic digestion. The initial development of PMF was made possible by the innovation of the matrix-assisted laser desorption/ionization (MALDI) soft ionization method during the late 1980s (63). MALDI represented a major breakthrough in protein chemistry, enabling large nonvolatile molecules, such as small proteins and peptides, to be ionized without fragmentation for downstream mass spectrometry. Coupled with a TOF analyzer, the MALDI-TOF mass spectrometry system is a robust, simple, and sensitive instrument with a large mass range (62) that is ideally suited for PMF. PMF works best on individual proteins and complex mixtures with reliable composition (64,65). Although most archaeological remains contain complex and variable protein mixtures, some collagenous tissues and residues are so dominated by COL1 that they can be analyzed by PMF. Using COL1 for taxonomic identification is referred to as ZooMS (1).

While the principles and concepts behind ZooMS are relatively straightforward, in practice the technique can be complicated by posttranslational and diagenetic modifications, the unavailability of taxonomically informative markers, and other factors. The first step in ZooMS analysis is to extract the collagen from its matrix. This step is the most variable as it depends upon the type of material, its preservation history, its previous treatment, and its ability to undergo destructive analysis. For mineralized tissues where destructive analysis can be used, dissolving the mineral matrix using hydrochloric acid is thought to be the most reliable method, especially for poorly preserved samples (1). For nonmineralized tissues, when less destructive analysis is desired or when the use of acid is problematic, alternative methods are also available. During the extraction process, COL1 is gelatinized using heat so that the primary amino acid structure is available for enzymatic digestion. Because soil humic acids and other base-soluble compounds can interfere with MALDI-TOF analysis, a brief incubation of the collagen in dilute sodium hydroxide (6,66) or other treatments (67) can be optionally applied to remove these compounds during extraction, thereby improving downstream spectral quality. At the end of the extraction process, the free collagen chains are suspended in a pH-neutral solution.

**Fig. 2.**
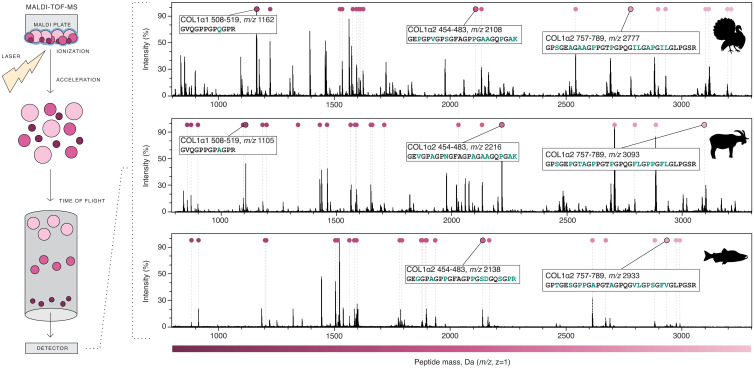
Steps of MALDI-TOF and representative collagen spectra. Digested collagen peptides (pink) are embedded in the matrix (blue) and ionized with a laser. Charged peptides (+1) are then accelerated through a TOF tube, where they separate by mass. The output of the detector is visualized as spectra, where time is converted to*m/z*based on calibration standards. Collagen spectra are shown for turkey (*M. gallopavo*), goat (*C. hircus*), and coho salmon (*O. kisutch*). Authenticated collagen peptide peaks are indicated by pink circles (Dataset S1*A*). Three taxonomically informative marker peptides are annotated, with*Insets*indicating the collagen chain, position,*m/z*, and sequence; amino acids that differ across taxa are highlighted in green. Note that although isoleucine (I) and leucine (L) differences are highlighted, they are not distinguishable by MALDI-TOF. Baseline correction, smoothing, and intensity normalization were performed in mMass (134). Adapted from ref.^42^. Data from refs.^21^,^140^, and^141^.

After extraction, the collagen is digested with a protease, typically trypsin, which cleaves the C-terminal peptide bonds of arginine (R) and lysine (K) residues and adds the mass of a water molecule to each peptide. This produces a series of collagen peptides that differ both in length and in mass between taxa (Fig. 1). The peptides are then acidified, purified with a C18 filter, and spotted onto an MALDI plate with a matrix, typically α-cyano-4-hydroxycinnamic acid, that cocrystalizes with the peptides (Fig. 2). The matrix is then excited with a laser, causing the peptides to vaporize and ionize with a +1 charge. Electromagnets direct the ions into a time-of-flight tube where they separate by mass, with the smallest peptides hitting the detector first and the largest hitting the detector last (Fig. 2). The resulting mass spectrum produced by the detector is then calibrated using standards to convert time into mass-to-charge ratios (*m/z*), and the observed peaks are ready for analysis (Fig. 2).

### Peptide Mass Fingerprints of Collagen and How They Are Used.

Interpreting a mass spectrum involves associating peaks with a given*m/z*to a specific peptide sequence. ZooMS typically uses MALDI-TOF to measure peptide masses between 800 and 3,500 Da, which correspond to peptides of ∼8 to 30 amino acids in length. Although theoretically all COL1 peptides in this mass range should appear in the PMF, in practice not all peptides are observed. Cross-linking, glycosylation, glycation, incomplete digestion, and poor ionization can contribute to the failure to observe predicted COL1 peptide peaks, as can unexpected PTMs and nonenzymatic peptide fragmentation.

Here, we show how this occurs in practice.Fig. 2shows the COL1 PMF of turkey (*Meleagris gallopavo*), goat (*Capra hircus*), and coho salmon (*Oncorhynchus kisutch*). The sequences of the COL1ɑ chains differ between these three species (Fig. 1), and this is reflected in the different peak positions of the peptides (Fig. 2). InFig. 2, peaks with masses corresponding to known collagen peptides are annotated with pink circles (Dataset S1*A*has details). Not all collagen peptides are taxonomically informative, but those that are taxonomically informative are called marker peptides. Three marker peptides—COL1ɑ1 508-519, COL1ɑ2 454-483, and COL1ɑ2 757-789 (nomenclature is after ref.^42^)—are highlighted. Although marker peptide COL1ɑ1 508-519 is not visible in the coho salmon spectrum, it has been previously observed and reported for this species (22).

**Fig. 3.**
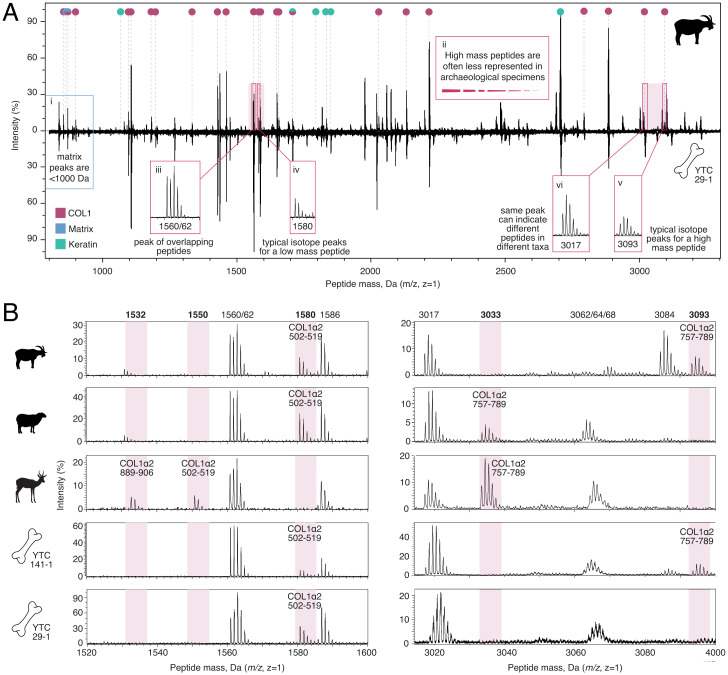
Interpreting a collagen peptide mass fingerprint. (*A*) Spectrum of a goat (*Upper*) and an archaeological unknown bone (YTC 29-1) from the site of Tepe Yahya, Iran (*Lower*). Colored circles indicate authenticated tryptic collagen peaks (pink), tryptic keratin peaks (green), and matrix peaks (blue). Peaks that could derive from different peptides of the same mass are indicated with both colors. (*A*,*i*) Matrix peaks are typically low mass (<1,000 Da) and overlap with short collagen peptides. (*A*,*ii*) High-mass peptides are often underrepresented. (*A*,*iii*) Peptides with the same or similar mass have overlapping peaks and may not be distinguishable, thus making them poor markers. (*A*,*iv*and*v*) Isotope distributions that derive from a single peptide peak differ for low- and high-mass peptides. (*vi*) Peaks that are not specific because they can derive from different peptides also make poor marker peptides. (*B*) Enhanced view of mass ranges from 1,520 to 1,600 Da (*Left*) and from 3,015 to 3,100 Da (*Right*) highlighted in*A*as well as from sheep (*O. aries*), springbok (*A. marsupialis*), and archaeological unknown bones YTC 141-1 and YTC 29-1. Collagen peak masses are indicated above the spectra with markers in bold and pink shading (Dataset S1*C*). Unknown bone YTC 141-1 has collagen markers 1,580 and 3,093 Da, indicating that it is a goat. Unknown YTC 29-1 has marker 1,580 Da, confirming that it belongs to Caprinae, but it lacks sufficient signal at higher-mass peptides to distinguish between sheep and goat. Data from ref.^141^.

Fig. 3illustrates some of the challenges in analyzing collagen PMFs. Besides collagen (pink circles), contaminant peaks are also present within ZooMS spectra, with the most common and abundant being keratins from skin and clothing (green circles) as well as matrix peaks (blue circles) and occasionally, autodigested trypsin peaks (68). Short COL1 peptides with masses <1,000 Da are rarely used as marker peptides because they frequently overlap with matrix peaks (Fig. 3*A*,*i*), and long, high-mass peptides are often less reliably visible in spectra (Fig. 3*A*,*ii*). Consequently, the most robust and useful marker peptides tend to be ∼1,000 to 3,100 Da in size (Dataset S1*B*). In vivo PTMs and diagenetiforms can complicate analysis by inducing mass shifts. However, only two are relevant for ZooMS, as most of these modifications are present at low levels or are spontaneous, and therefore they are not typically visible by MALDI-TOF MS. The most important PTM is hydroxyproline. Collagen spectra often contain peptide mixtures with different numbers of hydroxylated prolines, with each hydroxyproline causing a mass shift of +16 Da. In the example above, for instance, the sheep/cattle peptide COL1ɑ 757-789 typically produces two peaks: one at 3,017 Da with four hydroxyprolines and one at 3,033 Da with five hydroxyprolines (1). While rarely visible by MALDI-TOF MS, peptides with three and six hydroxyprolines are also present in bovids as shown by liquid chromatography-tandem mass spectrometry (LC-MS/MS) analysis (11). In addition to hydroxyprolines being abundant in mature collagen, recent studies have shown that stochastic gains and losses of hydroxyproline may also occur postmortem during diagenesis (54).

The most relevant diagenetiforms are the deamidation of glutamine (Q) to glutamic acid (E) and asparagine (N) to aspartic acid (D), which are both commonly observed in most archaeological proteins (53,69,70). Deamidation can occur through side chain hydrolysis or condensation reactions (53), resulting in a net +1 Da mass shift due to replacement of the amide with a carboxyl functional group. Depending on the amino acid sequence and degree of degradation, a given collagen peptide may contain zero, one, or more deamidated residues that result in overlapping peak distributions, and consequently multiple permutations must be taken into account during peak identification. Deamidation has been proposed as an indicator of relative age or as a way to identify intrusive samples (53,71–73). However, in practice, this use has been questioned (70,74) as it requires very large datasets to attempt, and even then the accuracy of assigning samples to age class is poor (75). It is likely that deamidation is strongly impacted by postmortem treatment, such as liming of parchments (76), by local depositional chemistry (74,75), and by choice of extraction method (77,78), which introduces variation that limits its straightforward application as an age indicator.

Finally, many high-abundance peaks in collagen PMFs are not marker peaks because they actually consist of two or more overlapping peptide peaks with identical or highly similar (differing by only ∼1 Da) masses (Fig. 3*A*,*iii*). For example, the peak at mass 3,017 Da shown inFig. 3*A*,*vi*has been found to be an unreliable marker peak because it can result either from the sheep/cattle variant of peptide COL1ɑ 757-789 containing four hydroxyprolines or from the highly conserved bovid peptide COL1ɑ2 90-130 with five hydroxyprolines (11). Such problematic peaks can sometimes be recognized because the pattern and intensity of their isotope peaks differ from those expected for low-mass (Fig. 3*A*,*iv*) or high-mass (Fig. 3*A*,*v*) peptides, but when the masses of the peptides are identical, overlapping peptides can only be identified using tandem mass spectrometry. For these reasons, it is essential to perform MS/MS analysis on candidate markers to determine their specificity and fidelity when developing or publishing new ZooMS markers for taxonomic identification (11,42).

Despite these challenges, however, when marker peptides are well chosen and spectra are carefully analyzed, ZooMS is a powerful tool for assigning taxonomy to unknown bones and other collagenous remains. An example of ZooMS identifications for two medium-sized mammal bones (YTC 29-1 and YTC 141-1) from the archaeological site of Tepe Yahya in Iran (79) is shown inFig. 3.Fig. 3*A*shows the full PMF of a reference goat (Fig. 3*A*,*Upper*) and YTC 29-1 (Fig. 3*A*,*Lower*). Of the 23 identified collagen peaks visible in the goat spectrum (pink circles), 16 are shared with other members of the Bovidae family. These peaks are also present in YTC 29-1 and YTC 141-1, indicating that the unknown bones derive from a bovid. The remaining seven collagen peaks are taxonomically variable within Bovidae, and of these, three are useful markers for distinguishing the medium-sized bovids possibly present at Tepe Yahya (Dataset S1*C*): goat, sheep (*Ovis aries*), and springbok (*Antidorcas marsupialis*) (10,23).Fig. 3*B*highlights the two mass windows where these diagnostic collagen peptides are visible (1,520 to 1,600 Da and 3,015 to 3,100 Da). Within the windows, a total of 13 peaks are present, only 5 of which correspond to the three diagnostic markers: COL1ɑ2 502-519 is present in sheep/goat at 1,580 Da and springbok at 1,550 Da; COL1ɑ2 757-789 is present at 3,033 Da in sheep/springbok and at 3,093 Da in goat; and COL1ɑ2 889-906 is present at 1,532 Da in springbok and at 1,560 Da in sheep/goat. Unfortunately, the peak at 1,560 Da overlaps with another collagen peptide and therefore is not informative. Archaeological bone YTC 141-1 has peaks 1,580 and 3,093 Da, allowing for an identification of goat. Archaeological bone YTC 29-1 has a peak at 1,580 Da, narrowing down the possibilities to sheep or goat, but it lacks a corresponding diagnostic peak at either 3,033 Da or 3,093 Da, suggesting that the COL1ɑ2 757-789 peptide was not detected due to a lack of preservation, a failure to ionize, or another taphonomic reason. Although a small peak may be discernible at 3,093 Da, it does not have an intensity value at least threefold above the background, thus making it unreliable to call. A signal to noise ratio of at least three is generally considered the limit of detection for MALDI-TOF, but higher limits should be used if the background noise is particularly strong (80,81). Consequently, YTC 29-1 can only be identified to sheep/goat.

## ZooMS: Methods and State of the Field

### Foundational Work on Mammals.

Prior to ZooMS, PMF was used to identify collagenous glues and paint binders from milk- or egg-based materials in artwork, with successful taxonomic discrimination between rabbit, cow, and fish glues (82,83). ZooMS was then developed to identify faunal skeletal remains using an initial set of seven markers from a comparative analysis of 32 mammals and four birds, including a variety of wild terrestrial and aquatic species, domesticates, and commensal small mammals (1). Early work focused on determining the utility of ZooMS for distinguishing particular species, such as sheep/goat (10) and mammoth/mastodon (84). As the field grew, the number of markers expanded to nine (Dataset S1*B*), and the technique was applied to a wider range of animals but still focused primarily on medium and large mammals (9,17,48,85). Over the past 5 years, the number of characterized mammals has dramatically increased and geographically diversified to include, for example, wild bovids (11), rodents (20,86), bats (20), and marsupials (87) (*SI Appendix*, Fig. S1). Recent work has also further explored multispecies collagen mixtures found in animal glues (88).

### Taxonomic Resolution and Expansion of the Marker Database.

Due to differing thermal and other functional constraints, collagen sequence variability differs substantially across major animal clades, such as birds, mammals, and fish (Dataset S1*D*and*SI Appendix*, Fig. S2), and this has important implications for the use of ZooMS to provide taxonomic identifications. Large mammals, for which collagen sequence data and ZooMS markers are most developed, are reliably identifiable to the family level (1,9), with examples of subfamily-level (e.g., cetaceans) and even genus-level (e.g., sheep/goat) separation being possible using the current nine standard markers. Recent work on identifying and confirming additional markers has increased the taxonomic resolution for bovids (11,23), elephantiformes (89), and some cervids (90), but current markers provide limited resolution for several groups where taxonomic discrimination would be useful, including camelids, felids, and many cervids. In small-bodied mammals, greater taxonomic resolution is often possible due to the larger variability of collagen sequences, with more cases of genus-level and even some species-level identifications (86,91).

From its earliest days, ZooMS was occasionally applied to nonmammals, but it is only recently that the method has been developed in earnest for birds (8), fish (21,22,92), amphibians (93), and reptiles (12,20,93). Fish have been shown to have the most collagen sequence variants (94,95) due to the relaxed functional constraints of their lower body temperature and buoyancy, as well as the presence of a distinct third ɑ-chain (COL1ɑ3) in most fishes (21,96). As such, fish have the highest potential for species-level taxonomic resolution (2,21,22,92,97,98). The high collagen sequence variability of fish is particularly beneficial for zooarchaeologists because fish bones are often difficult to identify due to their lack of morphologically identifiable features (99).

Preliminary investigations on the use of ZooMS to identify reptiles and amphibians have also achieved putative species-level identifications (12,93), although the low number of samples tested and the current lack of genetic data for many reptiles and amphibians make further work necessary. Interest in applying ZooMS to birds has lagged behind that of other groups, in part because the slower mutation rate of avian collagen makes it more difficult to distinguish related groups of birds (2,8), but it nevertheless remains useful for identifying key domesticates (8). For all faunal groups, improved taxonomic precision can be achieved when additional geographical, morphological, and archeological context information is taken into account to constrain possible ZooMS identifications. As more collagen sequences become available in genomic databases, the applicability of ZooMS to nonmammalian taxonomic groups is expected to improve.

### Development of Minimally Destructive and Noninvasive Sampling Protocols.

One advantage of ZooMS is that it can be applied to very small amounts of collagen, allowing for the use of minimally destructive and noninvasive sampling techniques. Mineralized samples and leather can be soaked overnight in an ammonia bicarbonate buffer and then heated briefly to extract soluble collagen (100). This method causes minimal observable damage to bone samples, which can then be dried, or subsequently reused for stable isotope or ancient DNA studies (4,100). Other minimally destructive and noninvasive sampling techniques rely on the triboelectric effect, in which friction between a plastic polymer and a protein creates electrostatic attraction that captures proteins on the surface of the polymer (28). This technique was first developed for parchments sampled using a polyvinyl chloride eraser (5). Since then, the triboelectric effect has also been used to successfully retrieve collagen from plastic sample bags and vials. This method is particularly suited to sampling worked bone artifacts (28,101) and has also been used to recover collagen from “empty” vials used in the lyophilization of collagen for isotopic analysis (16). Recently, a number of additional minimally destructive methods have been developed for art and cultural heritage materials. These include polishing films with grit (25), ethylene vinyl acetate films studded with strong cation and anion exchangers and C8/C18 resins (102), and enzyme functionalized films (103) and hydrogels (27).

While these minimally destructive sampling techniques are generally successful for well-preserved samples, they can be less effective for poorly preserved samples than conventional destructive methods and may result in fewer peaks (and therefore, lower taxonomic resolution) and lower-quality spectra. Nevertheless, they offer a number of advantages. Most importantly, they can be applied to rare or fragile artifacts where destructive sampling is not allowed. In addition, minimally destructive sampling and sample transport are also often easier to perform and can be carried out by nonspecialists (5,28,104). Finally, the sampling and extraction procedures of noninvasive techniques are generally faster than destructive methods because they eliminate the time-consuming acid digestion and neutralization steps.

### Expansion of PMF to Noncollagenous Proteins and Protein Mixtures.

Beyond collagen, other archaeological proteins and proteomes have also been explored for taxonomic identification using PMF. Like collagens, keratins and corneous beta-proteins (CBPs; formerly beta-keratins) are also highly ubiquitous structural proteins, with dozens of different characterized types found in mammalian epithelial cells as well as hair, wool, nails, quills, horn, baleen, feathers, turtle shells, and reptile scales (105–107). Although these tissues are not composed of one dominant protein, keratin and CBP mixtures can be taxonomically identified using PMF (108,109). Keratin markers have been developed for a few dozen mammal species (108–113), while CBP markers have only been developed for sea turtles (114,115). However, human and sheep keratins are also common contaminants in proteomics research, as human epithelial keratins are the primary constituents of airborne dust (116) and wool is a common component of clothing (68,117). This necessitates extreme caution when using markers that correspond to the masses of any of the tryptic peptides from sheep or human keratin. Although keratins and CBPs show great promise for further PMF development, research has been delayed by a lack of available sequence data and the historical nonuniformity of keratin nomenclature (118). However, with improved databases, achieving genus-level taxonomic resolution appears possible in many circumstances.

PMF can also be used for taxonomic identification of some complex protein mixtures, such as those found in eggshells and mollusk shells. Extraction pretreatment methods using sodium hypochlorite (NaOCl) can decontaminate and isolate the endogenous intracrystalline proteins before demineralization and digestion, thereby decreasing contamination (119,120). While advances in bird genome sequencing have aided the sequence determination of highly variable eggshell biomarkers across taxa (119), incomplete taxonomic availability of genomic data still limits the identification of most avian eggshell to order or family (121). Nevertheless, this technique has great potential for identifying avian taxa to the level of genus or species in the archaeological record (121–123) and is likely also suitable for identifying fossilized reptilian eggshells. While mollusk shells have long been known to contain organic compounds, including proteins, archaeological mollusk shell proteomes have only recently been explored through PMF (124–126). Although the database at present is very limited, shell matrix proteins are highly diverse and therefore have the potential to achieve genus- or species-level resolution (127).

PMF continues to be used in art conservation to identify noncollagenous paint binders, such as: caseins and beta lactoglobulin from milk; vitellogenins, apolipoproteins, and low-density lipoprotein receptors from egg yolk; and ovalbumin, ovotransferrin, and lysozyme from egg white (128). While some paint binders originate from a single source, identification of mixed sources is also possible with PMF, although LC-MS/MS is better suited for identifying mixtures of unknown composition (27,83). In some cases, the use of dual enzymes during digestion can provide enhanced resolution when using noncollagenous proteins (129).

Additional biomaterials that are known to contain proteins with at least some level of taxonomic variability include terrestrial snail shells (130), corals (131), and insect exoskeleton cuticles (132). These biomaterials preserve over archaeological timescales and show promise for future exploration, but they have only been minimally explored at present.

## Current Limitations in the Field

While there is an increasing interest in applying PMF—and ZooMS in particular—to investigate zooarchaeological remains, archaeological artifacts, cultural heritage materials, and works of art, there are also significant barriers to the adoption of the technique by new research groups. Below, we highlight solutions to improve data reporting, standardization, and accessibility.

First, there is currently no centralized repository of COL1 markers, MALDI-TOF reference spectra, or curated collagen sequences. Instead, each research group maintains their own internal reference datasets that need to be continuously updated with new publications. Additionally, while most markers have been verified by LC-MS/MS in their initial publication, some have not due to insufficient funds, problems of feasibility, or lack of corresponding genetic sequence data (92,97,133). In some cases, provisional markers have been later challenged or redefined once LC-MS/MS data became available (11). Without a centralized information hub, it can be difficult to track these changes, and this poses a significant obstacle for new researchers entering the field. Further complicating the learning process for new researchers is the fact that mass lists are often published with different levels of precision in*m/z*values, and multiple marker naming systems are currently in use (Dataset S1*B*), although there has recently been an attempt at standardization (42).

Second, while it is becoming more common for publications to make raw MALDI-TOF data available in public data repositories (11,21,23), the raw data for most early studies are not available, and even recent studies have not always made their data available. This reduces the replicability of ZooMS research and prevents data reanalysis as new markers are identified or existing markers are redefined in light of new evidence. As has been recently proposed for ancient protein studies using LC-MS/MS (69), new standards and guidelines are needed for publishing raw MALDI-TOF data.

Third, even when all publications are included, current ZooMS markers are still heavily biased toward large European mammals. Although other taxonomic groups are increasingly being studied, the number of published markers relative to the number of potential species remains low. In addition, because publicly available collagen gene and protein sequences are biased toward mammals (21), ZooMS continues to be available for only a small subset of the taxonomic groups of interest.

Fourth, there is a lack of centralized training resources and methodologies. Unlike other biomolecular specialties, the ZooMS community has not yet developed regular workshops for new researchers to learn about the field and its methods. Although mammal identification protocols (https://doi.org/10.17504/protocols.io.bzscp6aw) and detailed bench protocols for several common ZooMS methods (https://doi.org/10.17504/protocols.io.bf5djq26) have been recently published, most noninvasive protocols are only described in the methods sections of publications. There has also been a lack of investment in community software, with the most widely used open source software, mMass (134), no longer being developed or supported (http://www.mmass.org/). Recently, efforts have been made to keep mMass available and compatible with new computer operating systems (through the European Research Council FINDER project;https://github.com/dreamingspires/mMass), but additional community support is needed. In addition, most species identifications, even for high-throughput screening, are not automated. Several different methods of automation are currently being explored (135,136) but still remain in development.

Finally, ZooMS is fundamentally a tool to provide taxonomic identification. Currently, most ZooMS work focuses on developing markers, screening for specific species, or answering questions that involve a limited number of species. As ZooMS expands, there will need to be an expanded focus on situating ZooMS identifications within broader zooarchaeological frameworks and incorporating ZooMS data into standard zooarchaeological metrics, such as number of identified specimens, minimum number of individuals, and minimum number of elements (14,22). Because ZooMS can be conducted on fragmentary remains that would otherwise not be counted in these metrics, creating standards for how to report ZooMS results that allow comparison and integration with morphologically identified zooarchaeological datasets will be essential.

## Conclusion: ZooMS, the Next Decade

The first decade of ZooMS research has shown the potential for PMF techniques to revolutionize species identification of animal remains. However, to date, research has largely focused on method and marker development by a handful of research groups. Applications, by necessity, have frequently been limited in scope. Nevertheless, even with these limitations, ZooMS has had tremendous success, contributing to the discovery of the first Neanderthal–Denisovan child (137), identifying the earliest known domestic sheep in Africa (23), providing insights into the construction of the medieval York Gospels (138), and demonstrating that bone scraper technology for leatherworking has persisted for over 50,000 years (101).

The next major challenge for ZooMS will be to grow from a new method performed by highly specialized laboratories into a widely available and accessible technique with robustly supported software, centralized databases, and public data repositories. The number of research groups using ZooMS has steadily increased over the past decade, and while many of these laboratories are still actively involved in method and/or marker development, an increasing number of research groups are focusing on applied questions. The next decade of ZooMS promises an even greater expansion of applications by an even wider range of researchers, including by citizen science and K–12 educational groups (139). In order for ZooMS to successfully make the transition from a niche method developed by a handful of groups to a well-established technique with revolutionary applications, a community effort needs to be made to fund and establish an open-source marker database, stable data repositories, and training resources. The continued expansion of markers, aided by gene mining from large-scale genome sequencing projects, will allow for ZooMS to be applied more broadly in time and space and to more diverse taxa. Automation of spectral processing will increase the number of spectra that can be analyzed and provide a substantial body of data for zooarchaeologists and ecologists to analyze and interpret as they develop standardized ways to incorporate ZooMS results into established frameworks for faunal analysis and ecological reconstruction.

ZooMS will continue to be used as a screening tool for identifying taxa of interest, allowing researchers to find the proverbial needles in the haystack of millions of fragmentary and morphologically unidentifiable remains. Characterization of faunal assemblages will continue, but increasing geographical and temporal data will enable the tracking of ecological changes, the arrival of invasive species, and fluctuations in animal exploitation through space and time. Increasing markers for ecological indicator species, especially among small mammals, fish, and reptiles, will support more comprehensive ecological reconstructions of those ecosystems most vulnerable to climate change, thereby aiding in better understanding the factors that contribute to resilience and recovery. Targeted questions around archaeological artifacts and cultural heritage materials will allow for more nuanced interpretations of human–animal interactions and the cultural and technological choices made by our ancestors. Globally, at present there have been fewer than 50,000 samples analyzed by ZooMS. The next decade will be driven by the following question. What will we be able to achieve if we aim for a million ZooMS samples?

## Supplementary Material

Supplementary File

Supplementary File

## Data Availability

Peabody Museum accession codes for YTC 29-1 and YTC 114-1 are 986-7-60/22295 (https://collections.peabody.harvard.edu/objects/details/781283) and 986-7-60/22400 (https://collections.peabody.harvard.edu/objects/details/781388), respectively. MS spectra data have been deposited in Zenodo [salmon spectra,*O. kisutch*, York lot no. 15884 (https://doi.org/10.5281/zenodo.2649336) (140) and all other spectra (https://doi.org/10.5281/zenodo.5291648) (141)]. All other data are included in the manuscript and/or supporting information.

## References

[1] M. Buckley, M. Collins, J. Thomas-Oates, J. C. Wilson,Species identification by analysis of bone collagen using matrix-assisted laser desorption/ionisation time-of-flight mass spectrometry.Rapid Commun. Mass Spectrom. 23,3843–3854(2009). 19899187 10.1002/rcm.4316

[2] M. Buckley,“Zooarchaeology by mass spectrometry (ZooMS) collagen fingerprinting for the species identification of archaeological bone fragments”inZooarchaeology in Practice: Case Studies in Methodology and Interpretation in Archaeofaunal Analysis, C. M. Giovas, M. J. LeFebvre, Eds. (Springer International Publishing,2018), pp.227–247.

[3] A. N. Coutu, G. Whitelaw, P. le Roux, J. Sealy,Earliest evidence for the ivory trade in southern Africa: Isotopic and ZooMS analysis of seventh–tenth century and ivory from KwaZulu-Natal.Afr. Archaeol. Rev. 33,411–435(2016).

[4] I. C. C. von Holstein,Searching for Scandinavians in pre-Viking Scotland: Molecular fingerprinting of Early Medieval combs.J. Archaeol. Sci. 41,1–6(2014).

[5] S. Fiddyment,Animal origin of 13th-century uterine vellum revealed using noninvasive peptide fingerprinting.Proc. Natl. Acad. Sci. U.S.A. 112,15066–15071(2015). 26598667 10.1073/pnas.1512264112 PMC4679014

[6] J. A. Ebsen, K. Haase, R. Larsen, D. V. P. Sommer, L. Ø. Brandt,Identifying archaeological leather—discussing the potential of grain pattern analysis and zooarchaeology by mass spectrometry (ZooMS) through a case study involving medieval shoe parts from Denmark.J. Cult. Herit. 39,21–31(2019).

[7] D. P. Kirby, M. Buckley, E. Promise, S. A. Trauger, T. R. Holdcraft,Identification of collagen-based materials in cultural heritage.Analyst (Lond.) 138,4849–4858(2013). 23807214 10.1039/c3an00925d

[8] M. Eda, M. Morimoto, T. Mizuta, T. Inoué,ZooMS for birds: Discrimination of Japanese archaeological chickens and indigenous pheasants using collagen peptide fingerprinting.J. Archaeol. Sci. Rep. 34,102635(2020).

[9] M. Buckley,Species identification of archaeological marine mammals using collagen fingerprinting.J. Archaeol. Sci. 41,631–641(2014).

[10] M. Buckley,Distinguishing between archaeological sheep and goat bones using a single collagen peptide.J. Archaeol. Sci. 37,13–20(2010).

[11] A. Janzen,Distinguishing African bovids using Zooarchaeology by Mass Spectrometry (ZooMS): New peptide markers and insights into Iron Age economies in Zambia.PLoS One 16,e0251061(2021). 34003857 10.1371/journal.pone.0251061 PMC8130928

[12] V. L. Harvey,Preserved collagen reveals species identity in archaeological marine turtle bones from Caribbean and Florida sites.R. Soc. Open Sci. 6,191137(2019). 31824722 10.1098/rsos.191137 PMC6837194

[13] S. Brown,Identification of a new hominin bone from Denisova Cave, Siberia using collagen fingerprinting and mitochondrial DNA analysis.Sci. Rep. 6,23559(2016). 27020421 10.1038/srep23559 PMC4810434

[14] S. Brown,Zooarchaeology through the lens of collagen fingerprinting at Denisova Cave.Sci. Rep. 11,15457(2021). 34326389 10.1038/s41598-021-94731-2 PMC8322063

[15] S. Brown,The earliest Denisovans and their cultural adaptation.Nat. Ecol. Evol. 6,28–35(2021). 34824388 10.1038/s41559-021-01581-2 PMC7612221

[16] S. Charlton,Finding Britain’s last hunter-gatherers: A new biomolecular approach to “unidentifiable” bone fragments utilising bone collagen.J. Archaeol. Sci. 73,55–61(2016).

[17] F. Welker,Palaeoproteomic evidence identifies archaic hominins associated with the Châtelperronian at the Grotte du Renne.Proc. Natl. Acad. Sci. U.S.A. 113,11162–11167(2016). 27638212 10.1073/pnas.1605834113 PMC5056053

[18] M. Buckley, V. L. Harvey, A. T. Chamberlain,Species identification and decay assessment of Late Pleistocene fragmentary vertebrate remains from Pin Hole Cave (Creswell Crags, UK) using collagen fingerprinting.Boreas 46,402–411(2017).

[19] J. Ma,The Mammuthus-Coelodonta Faunal Complex at its southeastern limit: A biogeochemical paleoecology investigation in Northeast Asia.Quat. Int. 591,93–106(2021).

[20] V. L. Harvey,Interpreting the historical terrestrial vertebrate biodiversity of Cayman Brac (Greater Antilles, Caribbean) through collagen fingerprinting.Holocene 29,531–542(2019).

[21] K. K. Richter,What’s the catch? Archaeological application of rapid collagen-based species identification for Pacific Salmon.J. Archaeol. Sci. 116,105116(2020).

[22] V. L. Harvey, L. Daugnora, M. Buckley,Species identification of ancient Lithuanian fish remains using collagen fingerprinting.J. Archaeol. Sci. 98,102–111(2018).

[23] A. N. Coutu,Palaeoproteomics confirm earliest domesticated sheep in southern Africa ca. 2000 BP.Sci. Rep. 11,6631(2021). 33758223 10.1038/s41598-021-85756-8 PMC7988125

[24] S. Fiddyment,Girding the loins? Direct evidence of the use of a medieval English parchment birthing girdle from biomolecular analysis.R. Soc. Open Sci. 8,202055(2021). 33959357 10.1098/rsos.202055 PMC8074970

[25] D. P. Kirby, A. Manick, R. Newman,Minimally invasive sampling of surface coatings for protein identification by peptide mass fingerprinting: A case study with photographs.J. Am. Inst. Conserv. 59,235–245(2020).

[26] S. P. Ashby, A. N. Coutu, S. M. Sindbaek,Urban networks and arctic outlands: Craft specialists and reindeer antler in Viking towns.Eur. J. Archaeol. 18,679–704(2015).

[27] C. D. Calvano, E. Rigante, R. A. Picca, T. R. I. Cataldi, L. Sabbatini,An easily transferable protocol for in-situ quasi-non-invasive analysis of protein binders in works of art.Talanta 215,120882(2020). 32312431 10.1016/j.talanta.2020.120882

[28] K. McGrath,Identifying archaeological bone via non-destructive ZooMS and the materiality of symbolic expression: Examples from Iroquoian bone points.Sci. Rep. 9,11027(2019). 31363122 10.1038/s41598-019-47299-x PMC6667708

[29] A. Desmond,ZooMS identification of bone tools from the North African Later Stone Age.J. Archaeol. Sci. 98,149–157(2018).

[30] A. Wagner,Whale bone puzzles: Reconstructing and identifying historical whale skeletons using archive records, osteology, and zooarchaeology by Mass Spectrometry (ZooMS).J. Conserv. Mus. Stud. 18,1–12(2020).

[31] R. P. Boot-Handford, D. S. Tuckwell,Fibrillar collagen: The key to vertebrate evolution? A tale of molecular incest.BioEssays 25,142–151(2003). 12539240 10.1002/bies.10230

[32] S. Ricard-Blum,The collagen family.Cold Spring Harb. Perspect. Biol. 3,a004978(2011). 21421911 10.1101/cshperspect.a004978 PMC3003457

[33] K. Henriksen, M. A. Karsdal,“Type I collagen”inBiochemistry of Collagens, Laminins and Elastin: Structure, Function and Biomarkers, M. A. Karsdal, D. J. Leeming, K. Henriksen, A.-C. Bay-Jensen, Eds. (Elsevier,2016), pp.1–11.

[34] T. Nagai, M. Izumi, M. Ishii,Fish scale collagen. Preparation and partial characterization.Int. J. Food Sci. Technol. 39,239–244(2004).

[35] Y. Chang, P.-Y. Chen,Hierarchical structure and mechanical properties of snake (*Naja atra*) and turtle (*Ocadia sinensis*) eggshells.Acta Biomater. 31,33–49(2016). 26607769 10.1016/j.actbio.2015.11.040

[36] I. Mikšík, P. Sedláková, K. Lacinová, S. Pataridis, A. Eckhardt,Determination of insoluble avian eggshell matrix proteins.Anal. Bioanal. Chem. 397,205–214(2010). 19998026 10.1007/s00216-009-3326-3

[37] O. B. A. Agbaje, S. C. George, Z. Zhang, G. A. Brock, L. E. Holmer,Characterization of organophosphatic brachiopod shells: Spectroscopic assessment of collagen matrix and biomineral components.RSC Advances 10,38456–38467(2020). 35517531 10.1039/d0ra07523j PMC9057340

[38] M. D. Shoulders, R. T. Raines,Collagen structure and stability.Annu. Rev. Biochem. 78,929–958(2009). 19344236 10.1146/annurev.biochem.77.032207.120833 PMC2846778

[39] S. Kimura, Y. Ohno,Fish type I collagen: Tissue-specific existence of two molecular forms, (α1)2α2 and α1α2α3, in Alaska pollack.Comp. Biochem. Physiol. B 88,409–413(1987).

[40] G. Némethy, H. A. Scheraga,Stabilization of collagen fibrils by hydroxyproline.Biochemistry 25,3184–3188(1986). 3730354 10.1021/bi00359a016

[41] J. Bella,Collagen structure: New tricks from a very old dog.Biochem. J. 473,1001–1025(2016). 27060106 10.1042/BJ20151169

[42] S. Brown, K. Douka, M. J. Collins, K. K. Richter,On the standardization of ZooMS nomenclature.J. Proteomics 235,104041(2021). 33160104 10.1016/j.jprot.2020.104041

[43] S. Perumal, O. Antipova, J. P. R. O. Orgel,Collagen fibril architecture, domain organization, and triple-helical conformation govern its proteolysis.Proc. Natl. Acad. Sci. U.S.A. 105,2824–2829(2008). 18287018 10.1073/pnas.0710588105 PMC2268544

[44] Z. Xu, W. Zhao, Z. Wang, Y. Yang, N. Sahai,Structure analysis of collagen fibril at atomic-level resolution and its implications for intra-fibrillar transport in bone biomineralization.Phys. Chem. Chem. Phys. 20,1513–1523(2018). 29260165 10.1039/c7cp05261h

[45] R. C. Dobberstein,Archaeological collagen: Why worry about collagen diagenesis? Archaeol. Anthropol. Sci. 1,31–42(2009).

[46] C. Kendall, A. M. H. Eriksen, I. Kontopoulos, M. J. Collins, G. Turner-Walker,Diagenesis of archaeological bone and tooth.Palaeogeogr. Palaeoclimatol. Palaeoecol. 491,21–37(2018).

[47] G. J. van Klinken,Bone collagen quality indicators for palaeodietary and radiocarbon measurements.J. Archaeol. Sci. 26,687–695(1999).

[48] N. Rybczynski,Mid-Pliocene warm-period deposits in the High Arctic yield insight into camel evolution.Nat. Commun. 4,1550(2013). 23462993 10.1038/ncomms2516 PMC3615376

[49] T. F. G. Higham, R. M. Jacobi, C. Bronk Ramsey,AMS radiocarbon dating of ancient bone using ultrafiltration.Radiocarbon 48,179–195(2006).

[50] T. Higham,The timing and spatiotemporal patterning of Neanderthal disappearance.Nature 512,306–309(2014). 25143113 10.1038/nature13621

[51] A. M. Child,Towards and understanding of the microbial decomposition of archaeological bone in the burial environment.J. Archaeol. Sci. 22,165–174(1995).

[52] G. Turner-Walker,“The chemical and microbial degradation of bones and teeth”inAdvances in Human Palaeopathology, R. Pinhasi, S. Mays, Eds. (Wiley,2008), pp.3–29.

[53] N. L. van Doorn, J. Wilson, H. Hollund, M. Soressi, M. J. Collins,Site-specific deamidation of glutamine: A new marker of bone collagen deterioration.Rapid Commun. Mass Spectrom. 26,2319–2327(2012). 22956324 10.1002/rcm.6351

[54] T. P. Cleland, E. R. Schroeter, M. H. Schweitzer,Biologically and diagenetically derived peptide modifications in moa collagens.Proc. Biol. Sci. 282,20150015(2015). 25972464 10.1098/rspb.2015.0015 PMC4455796

[55] F. Brock,Reliability of nitrogen content (%N) and carbon: Nitrogen atomic ratios (C:N) as indicators of collagen preservation suitable for radiocarbon dating.Radiocarbon 54,879–886(2012).

[56] E. J. Guiry, P. Szpak,Quality control for modern bone collagen stable carbon and nitrogen isotope measurements.Methods Ecol. Evol. 11,1049–1060(2020).

[57] Ö. Kalenderer, A. Turgut,“Bone”inMusculoskeletal Research and Basic Science, F. Korkusuz, Ed. (Springer,2016), pp.303–321.

[58] J. D. Currey,The structure and mechanics of bone.J. Mater. Sci. 47,41–54(2012).

[59] H. P. Schwarcz, H. Nahal,Theoretical and observed C/N ratios in human bone collagen.J. Archaeol. Sci. 131,105396(2021).

[60] N. Wang,Testing the efficacy and comparability of ZooMS protocols on archaeological bone.J. Proteomics 233,104078(2021). 33338688 10.1016/j.jprot.2020.104078

[61] N. Procopio, R. J. A. Hopkins, V. L. Harvey, M. Buckley,Proteome variation with collagen yield in ancient bone.J. Proteome Res. 20,1754–1769(2021). 33529527 10.1021/acs.jproteome.0c01014 PMC7944572

[62] R. Aebersold, D. R. Goodlett,Mass spectrometry in proteomics.Chem. Rev. 101,269–295(2001). 11712248 10.1021/cr990076h

[63] M. Karas, D. Bachmann, U. Bahr, F. Hillenkamp,Matrix-assisted ultraviolet laser desorption of non-volatile compounds.Int. J. Mass Spectrom. Ion Process. 78,53–68(1987).

[64] A. Wieser, L. Schneider, J. Jung, S. Schubert,MALDI-TOF MS in microbiological diagnostics-identification of microorganisms and beyond (mini review).Appl. Microbiol. Biotechnol. 93,965–974(2012). 22198716 10.1007/s00253-011-3783-4

[65] W. Yan,Principal component analysis of MALDI-TOF MS of whole-cell foodborne pathogenic bacteria.Anal. Biochem. 592,113582(2020). 31935357 10.1016/j.ab.2020.113582

[66] P. Szpak, K. Krippner, M. P. Richards,Effects of sodium hydroxide treatment and ultrafiltration on the removal of humic contaminants from archaeological bone.Int. J. Osteoarchaeol. 27,1070–1077(2017).

[67] S. Oonk, E. Cappellini, M. J. Collins,Soil proteomics: An assessment of its potential for archaeological site interpretation.Org. Geochem. 50,57–67(2012).

[68] B. O. Keller, J. Sui, A. B. Young, R. M. Whittal,Interferences and contaminants encountered in modern mass spectrometry.Anal. Chim. Acta 627,71–81(2008). 18790129 10.1016/j.aca.2008.04.043

[69] J. Hendy,A guide to ancient protein studies.Nat. Ecol. Evol. 2,791–799(2018). 29581591 10.1038/s41559-018-0510-x

[70] E. R. Schroeter, T. P. Cleland,Glutamine deamidation: An indicator of antiquity, or preservational quality? Rapid Commun. Mass Spectrom. 30,251–255(2016). 26689157 10.1002/rcm.7445

[71] V. Sinet-Mathiot,Combining ZooMS and zooarchaeology to study Late Pleistocene hominin behaviour at Fumane (Italy).Sci. Rep. 9,12350(2019). 31451791 10.1038/s41598-019-48706-z PMC6710433

[72] F. Welker,Variations in glutamine deamidation for a Châtelperronian bone assemblage as measured by peptide mass fingerprinting of collagen.Sci. Technol. Archaeol. Res. 3,15–27(2017).

[73] A. Ramsøe,DeamiDATE 1.0: Site-specific deamidation as a tool to assess authenticity of members of ancient proteomes.J. Archaeol. Sci. 115,105080(2020).

[74] M. Pal Chowdhury,Collagen deamidation in archaeological bone as an assessment for relative decay rates.Archaeometry 61,1382–1398(2019).

[75] S. Brown,Examining collagen preservation through glutamine deamidation at Denisova Cave.J. Archaeol. Sci. 133,105454(2021).

[76] S. Doherty, M. M. Alexander, J. Vnouček, J. Newton, M. J. Collins,Measuring the impact of parchment production on skin collagen stable isotope (δ13C and δ15N) values.Sci. Technol. Archaeol. Res. 7,1–12(2021).

[77] J. P. Simpson,The effects of demineralisation and sampling point variability on the measurement of glutamine deamidation in type I collagen extracted from bone.J. Archaeol. Sci. 69,29–38(2016).

[78] N. Procopio, M. Buckley,Minimizing laboratory-induced decay in bone proteomics.J. Proteome Res. 16,447–458(2017). 28152590 10.1021/acs.jproteome.6b00564

[79] R. H. Meadow,Animal Exploitation in Prehistoric Southeastern Iran: Faunal Remains from Tepe Yahya and Tepe Gaz Tavila-R37, 5500-3000 B.C.(Harvard University,1986).

[80] T. L. Y. Sheehan, R. A. Yost,What’s the most meaningful standard for mass spectrometry: Instrument detection limit or signal-to-noise ratio.Curr Trends Mass Spectrom 13,16–22(2015).

[81] J. V. Johnson, R. A. Yost,Tandem mass spectrometry for trace analysis.Anal. Chem. 57,758A–768A(1985).3993949

[82] R. Hynek, S. Kuckova, J. Hradilova, M. Kodicek,Matrix-assisted laser desorption/ionization time-of-flight mass spectrometry as a tool for fast identification of protein binders in color layers of paintings.Rapid Commun. Mass Spectrom. 18,1896–1900(2004). 15329854 10.1002/rcm.1570

[83] S. Kuckova, R. Hynek, M. Kodicek,Identification of proteinaceous binders used in artworks by MALDI-TOF mass spectrometry.Anal. Bioanal. Chem. 388,201–206(2007). 17340079 10.1007/s00216-007-1206-2

[84] M. Buckley, N. Larkin, M. Collins,Mammoth and Mastodon collagen sequences; survival and utility.Geochim. Cosmochim. Acta 75,2007–2016(2011).

[85] F. Welker, M. Soressi, W. Rendu, J.-J. Hublin, M. Collins,Using ZooMS to identify fragmentary bone from the Late Middle/Early Upper Palaeolithic sequence of Les Cottés, France.J. Archaeol. Sci. 54,279–286(2015).

[86] M. Buckley, M. Gu, S. Shameer, S. Patel, A. T. Chamberlain,High-throughput collagen fingerprinting of intact microfaunal remains; a low-cost method for distinguishing between murine rodent bones.Rapid Commun. Mass Spectrom. 30,805–812(2016). 27408951 10.1002/rcm.7483 PMC4831026

[87] M. Buckley, R. Cosgrove, J. Garvey, G. J. Prideaux,Identifying remains of extinct kangaroos in Late Pleistocene deposits using collagen fingerprinting.J. Quat. Sci. 32,653–660(2017).

[88] J. Vnouček,“The parchment of the Vienna Genesis: Characteristics and manufacture”inThe Vienna Genesis: Material Analysis and Conservation of a Late Antique Illuminated Manuscript on Purple Parchment, C. Hofmann, Ed. (Boehlau Verlag GmbH & Co. KG,2020), pp.35–70.

[89] M. Buckley, O. P. Recabarren, C. Lawless, N. García, M. Pino,A molecular phylogeny of the extinct South American gomphothere through collagen sequence analysis.Quat. Sci. Rev. 224,105882(2019).

[90] T. Z. T. Jensen,An integrated analysis of Maglemose bone points reframes the Early Mesolithic of Southern Scandinavia.Sci. Rep. 10,17244(2020). 33057088 10.1038/s41598-020-74258-8 PMC7560828

[91] M. Buckley, J. Herman,Species identification of Late Pleistocene bat bones using collagen fingerprinting.Int. J. Osteoarchaeol. 29,1051–1059(2019).

[92] T. Rick, V. L. Harvey, M. Buckley,Collagen fingerprinting and the Chumash billfish fishery, Santa Barbara Channel, California, USA.Archaeol. Anthropol. Sci. 11,6639–6648(2019).

[93] M. Buckley, M. Cheylan,Collagen fingerprinting for the species identification of archaeological amphibian remains.Boreas 49,709–717(2020).

[94] A. P. Martin, S. R. Palumbi,Body size, metabolic rate, generation time, and the molecular clock.Proc. Natl. Acad. Sci. U.S.A. 90,4087–4091(1993). 8483925 10.1073/pnas.90.9.4087 PMC46451

[95] N. Takezaki,Global rate variation in bony vertebrates.Genome Biol. Evol. 10,1803–1815(2018). 29931060 10.1093/gbe/evy125 PMC6055543

[96] J. Pasquier,Gene evolution and gene expression after whole genome duplication in fish: The PhyloFish database.BMC Genomics 17,368(2016). 27189481 10.1186/s12864-016-2709-z PMC4870732

[97] K. K. Richter,Fish’n chips: ZooMS peptide mass fingerprinting in a 96 well plate format to identify fish bone fragments.J. Archaeol. Sci. 38,1502–1510(2011).

[98] M. Buckley, M. Pinsonneault, C. Brassey, B. Rolett,High-throughput microCT and ZooMS collagen fingerprinting of Scombrid bone from the Marquesas Islands.J. Archaeol. Sci. 136,105475(2021).

[99] K. W. Gobalet,A critique of faunal analysis: Inconsistency among experts in blind tests.J. Archaeol. Sci. 28,377–386(2001).

[100] N. L. van Doorn, H. Hollund, M. J. Collins,A novel and non-destructive approach for ZooMS analysis: Ammonium bicarbonate buffer extraction.Archaeol. Anthropol. Sci. 3,281–289(2011).

[101] N. L. Martisius,Non-destructive ZooMS identification reveals strategic bone tool raw material selection by Neandertals.Sci. Rep. 10,7746(2020). 32385291 10.1038/s41598-020-64358-w PMC7210944

[102] G. Zilberstein, S. Zilberstein, U. Maor, P. G. Righetti,Surface analysis of ancient parchments via the EVA film: The Aleppo Codex.Anal. Biochem. 604,113824(2020). 32649932 10.1016/j.ab.2020.113824

[103] P. Cicatiello,Minimally invasive and portable method for the identification of proteins in ancient paintings.Anal. Chem. 90,10128–10133(2018). 30063323 10.1021/acs.analchem.8b01718

[104] S. Fiddyment,So you want to do biocodicology? A field guide to the biological analysis of parchment.Herit. Sci. 7,35(2019).

[105] D. Dhouailly,Getting to the root of scales, feather and hair: As deep as odontodes? Exp. Dermatol. 28,503–508(2019). 28603898 10.1111/exd.13391

[106] L. Eckhart, F. Ehrlich,Evolution of trichocyte keratins.Adv. Exp. Med. Biol. 1054,33–45(2018). 29797266 10.1007/978-981-10-8195-8_4

[107] R. C. Marshall, D. F. Orwin, J. M. Gillespie,Structure and biochemistry of mammalian hard keratin.Electron Microsc. Rev. 4,47–83(1991). 1714783 10.1016/0892-0354(91)90016-6

[108] C. Solazzo,Characterisation of novel α-keratin peptide markers for species identification in keratinous tissues using mass spectrometry.Rapid Commun. Mass Spectrom. 27,2685–2698(2013). 24591030 10.1002/rcm.6730

[109] C. Solazzo, W. Fitzhugh, S. Kaplan, C. Potter, J. M. Dyer,Molecular markers in keratins from Mysticeti whales for species identification of baleen in museum and archaeological collections.PLoS One 12,e0183053(2017). 28854252 10.1371/journal.pone.0183053 PMC5576650

[110] C. Solazzo,Characterizing historical textiles and clothing with proteomics.Conserv. Patrim. 31,97–114(2019).

[111] C. Solazzo,Proteomics and Coast Salish blankets: A tale of shaggy dogs? Antiquity 85,1418–1432(2011).

[112] C. Solazzo,Follow-up on the characterization of peptidic markers in hair and fur for the identification of common North American species.Rapid Commun. Mass Spectrom. 31,1375–1384(2017). 28600869 10.1002/rcm.7923

[113] K. Hollemeyer, W. Altmeyer, E. Heinzle, C. Pitra,Matrix-assisted laser desorption/ionization time-of-flight mass spectrometry combined with multidimensional scaling, binary hierarchical cluster tree and selected diagnostic masses improves species identification of Neolithic keratin sequences from furs of the Tyrolean Iceman Oetzi.Rapid Commun. Mass Spectrom. 26,1735–1745(2012). 22777774 10.1002/rcm.6277

[114] S. O’Connor, C. Solazzo, M. Collins,Advances in identifying archaeological traces of horn and other keratinous hard tissues.Stud. Conserv. 60,393–417(2015).

[115] C. Solazzo, J. Soulat, T. Cleland,Creation of a peptide database of corneous beta-proteins of marine turtles for the identification of tortoiseshell: Archaeological combs as case study.R. Soc. Open Sci. 8,201857(2021). 33972868 10.1098/rsos.201857 PMC8074788

[116] K. Fox, E. Castanha, A. Fox, C. Feigley, D. Salzberg,Human K10 epithelial keratin is the most abundant protein in airborne dust of both occupied and unoccupied school rooms.J. Environ. Monit. 10,55–59(2008). 18175017 10.1039/b714802j

[117] K. Hodge, S. T. Have, L. Hutton, A. I. Lamond,Cleaning up the masses: Exclusion lists to reduce contamination with HPLC-MS/MS.J. Proteomics 88,92–103(2013). 23501838 10.1016/j.jprot.2013.02.023 PMC3714598

[118] H. Gong,Emerging issues with the current keratin-associated protein nomenclature.Int. J. Trichology 2,104–105(2010). 21712897 10.4103/0974-7753.77519 PMC3107952

[119] S. Presslee,The identification of archaeological eggshell using peptide markers.Sci. Technol. Archaeol. Res. 3,89–99(2017).

[120] J. R. M. Stewart,Making eggshell visible in the archaeological record.J. Archaeol. Sci. 40,1797–1804(2013).

[121] B. Demarchi,Birds of prey and humans in prehistoric Europe: A view from El Mirón Cave, Cantabria (Spain).J. Archaeol. Sci. Rep. 24,244–252(2019).

[122] B. Demarchi, S. Presslee, J. Sakalauskaite, R. Fischer, J. Best,The role of birds at Çatalhöyük revealed by the analysis of eggshell.Quat. Int. 543,50–60(2020).

[123] M. Maltby, M. Allen, J. Best, B. T. Fothergill, B. Demarchi,Counting Roman chickens: Multidisciplinary approaches to human-chicken interactions in Roman Britain.J. Archaeol. Sci. Rep. 19,1003–1015(2018).

[124] J. Sakalauskaite,‘Palaeoshellomics’ reveals the use of freshwater mother-of-pearl in prehistory.eLife 8,8(2019). 10.7554/eLife.45644 PMC6542584 31060688

[125] J. Sakalauskaite, F. Marin, B. Pergolizzi, B. Demarchi,Shell palaeoproteomics: First application of peptide mass fingerprinting for the rapid identification of mollusc shells in archaeology.J. Proteomics 227,103920(2020). 32712371 10.1016/j.jprot.2020.103920

[126] J. Sakalauskaite,The shell matrix of the European thorny oyster, Spondylus gaederopus: Microstructural and molecular characterization.J. Struct. Biol. 211,107497(2020). 32220629 10.1016/j.jsb.2020.107497

[127] F. Marin,Mollusc shellomes: Past, present and future.J. Struct. Biol. 212,107583(2020). 32721585 10.1016/j.jsb.2020.107583

[128] S. Kuckova, P. Cejnar, J. Santrucek, R. Hynek,Characterization of proteins in cultural heritage using MALDI–TOF and LC–MS/MS mass spectrometric techniques.Phys Sci. Rev. 4,20180011(2019).

[129] C. D. Calvano, E. C. L. Rigante, T. R. I. Cataldi, L. Sabbatini,*In situ*hydrogel extraction with dual-enzyme digestion of proteinaceous binders: The key for reliable mass spectrometry investigations of artworks.Anal. Chem. 92,10257–10261(2020). 32648736 10.1021/acs.analchem.0c01898

[130] K. Mann, D. J. Jackson,Characterization of the pigmented shell-forming proteome of the common grove snail*Cepaea nemoralis*.BMC Genomics 15,249(2014). 24684722 10.1186/1471-2164-15-249 PMC4023409

[131] J. L. Drake, J. P. Whitelegge, D. K. Jacobs,First sequencing of ancient coral skeletal proteins.Sci. Rep. 10,19407(2020). 33173075 10.1038/s41598-020-75846-4 PMC7655939

[132] V. Masson, K. Arafah, S. Voisin, P. Bulet,Comparative proteomics studies of insect cuticle by tandem mass spectrometry: Application of a novel proteomics approach to the pea aphid cuticular proteins.Proteomics 18,18(2018). 10.1002/pmic.201700368 29327416

[133] J. Bradfield, T. Forssman, L. Spindler, A. R. Antonites,Identifying the animal species used to manufacture bone arrowheads in South Africa.Archaeol. Anthropol. Sci. 11,2419–2434(2019).

[134] M. Strohalm, M. Hassman, B. Kosata, M. Kodícek,mMass data miner: An open source alternative for mass spectrometric data analysis.Rapid Commun. Mass Spectrom. 22,905–908(2008). 18293430 10.1002/rcm.3444

[135] S. Hickinbotham, S. Fiddyment, T. L. Stinson, M. J. Collins,How to get your goat: Automated identification of species from MALDI-ToF spectra.Bioinformatics 36,3719–3725(2020). 32176274 10.1093/bioinformatics/btaa181 PMC7320604

[136] M. Gu, M. Buckley,Semi-supervised machine learning for automated species identification by collagen peptide mass fingerprinting.BMC Bioinformatics 19,241(2018). 29940843 10.1186/s12859-018-2221-3 PMC6019507

[137] V. Slon,The genome of the offspring of a Neanderthal mother and a Denisovan father.Nature 561,113–116(2018). 30135579 10.1038/s41586-018-0455-x PMC6130845

[138] M. D. Teasdale,The York Gospels: A 1000-year biological palimpsest.R. Soc. Open Sci. 4,170988(2017). 29134095 10.1098/rsos.170988 PMC5666278

[139] L. Ø. Brandt, M. R. Lillemark, M. Rytter, M. J. Collins, A. P. Tøttrup,Next Generation Lab puts Denmark’s past in the hands of its future.Antiquity 96,221–228.

[140] K. K. Richter, K. McGrath. MALDI-TOF spectra of archaeological (Oncorhynchus) and modern (Salmo salar) bone collagen. Zenodo. 10.5281/zenodo.2649336. Deposited 23 April 2019.

[141] K. K. Richter, M. Codlin, M. Seabrook, C. Warinner. ZooMS spectra. Zenodo. 10.5281/zenodo.5291648. Deposited 1 August 2021.

