# Supplementary material for: A primer for ZooMS applications in archaeology
Source: Proc Natl Acad Sci U S A. 2022 May 10;119(20):e2109323119. doi: 10.1073/pnas.2109323119 (PMC9171758; doi:10.1073/pnas.2109323119)
Supplement: Supplementary File [file pnas.2109323119.sapp.pdf]

Supplementary Information for

A primer for ZooMS applications in archaeology

Kristine Korzow Richter, Maria Codlin, Melina Seabrook, Christina Warinner

Correspondence to:

Christina Warinner	Kristine Korzow Richter
warinner@fas.harvard.edu	kkrichter@palaeome.org

This PDF file includes:

Supplementary Figures S1-S2
Supplementary Note

Supplementary Figures

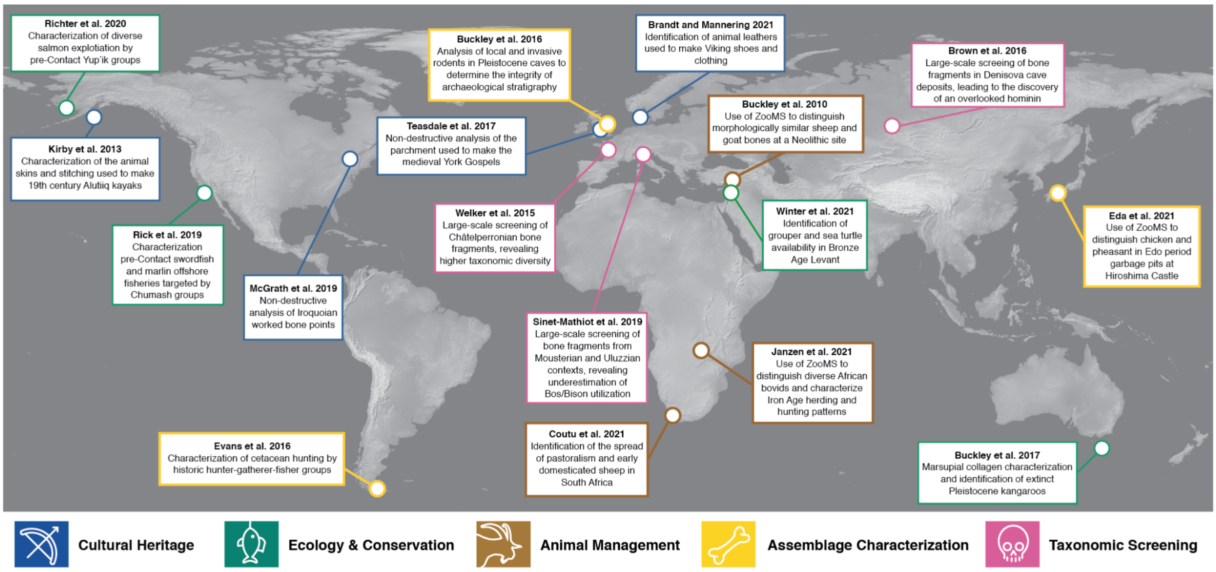

Figure S1. Growing use and applications of ZooMS. Since its development in 2009 (1), the number and scope of ZooMS applications has rapidly expanded. Today, ZooMS is used for a wide range of purposes in archaeology, ecology, and cultural heritage, including characterizing faunal assemblages, identifying animal management strategies, screening for taxa of interest, characterizing current and past ecologies, and supporting cultural heritage preservation. Icons from https://openmoji.org.

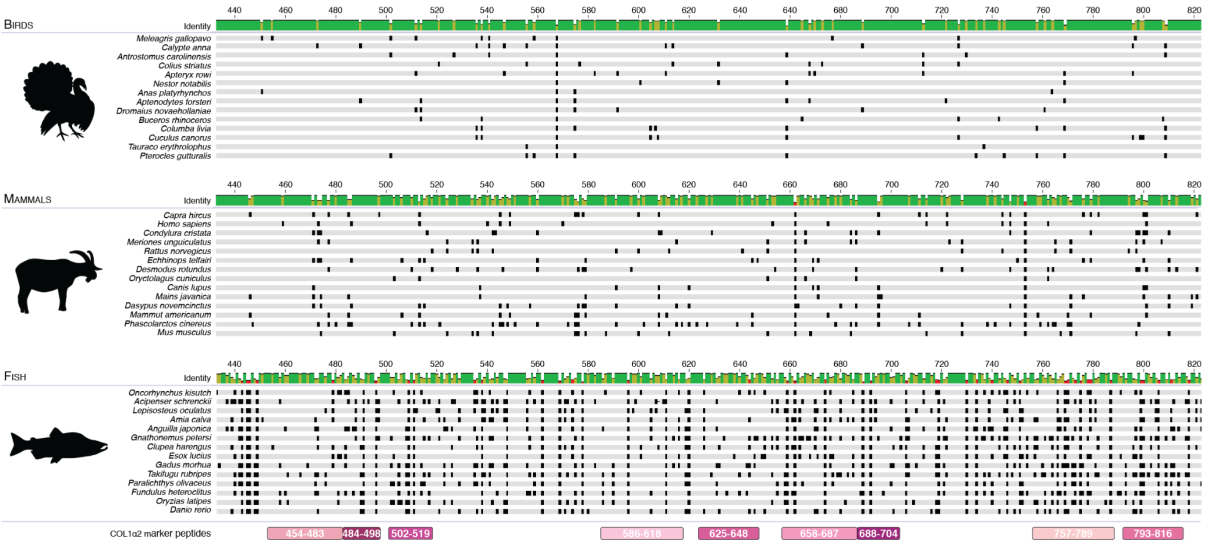

Figure S2. Comparison of collagen sequence diversity across birds, mammals, and fish. Alignment depicts COL1α2 432-824 (nomenclature from (2)) from a diverse set of birds, mammals, and fish (see Dataset S1D). Amino acid sequence variants deviating from taxonomic group consensus are marked in black and marker peptides are shown in pink. Sequences were aligned using Geneious 2019.0.4 (Biomatters Ltd.).

Supplementary Note

Since 2018, when the last comprehensive reviews of ZooMS were written (3–5), there has been explosive growth in the number of ZooMS related publications (2). The application of ZooMS to faunal assemblages for the characterization and identification of animal use and management strategies (6–9) has allowed a wide range of topics to be explored, including domestic herd management (10–17), choices relating to secondary product use (18–21), exploitation of wild species (22–26), and the appearance of commensal species (27–30). In conjunction with assemblage characterization, ZooMS has also been productively applied as a screening tool for identifying specific taxa of interest (31–37). ZooMS is also useful for archaeological and culture heritage conservation, and can be used to identify worked bone and composite artefacts, including bone points and arrowheads (38–45), daggers (46), rings (47), combs (48), worked antler (49), leather items (50, 51), parchment (18–21, 52, 53), works of art (54–58), and other organic artifacts (59–61). Although less developed, ZooMS has great potential to allow the characterization of current and past ecologies within both terrestrial (62–64) and aquatic ecosystems (65–70), with particular implications for conservation and identification of the trade of illicit animal products (15, 22, 71). ZooMS applications span large chronological periods and geographical ranges, enabling the method to play an important role in bridging diverse disciplines (Fig. S1).

References

1. M. Buckley, M. Collins, J. Thomas-Oates, J. C. Wilson, Species identification by analysis of bone collagen using matrix-assisted laser desorption/ionisation time-of-flight mass spectrometry. *Rapid Commun. Mass Spectrom.* **23**, 3843–3854 (2009).

2. S. Brown, K. Douka, M. J. Collins, K. K. Richter, On the standardization of ZooMS nomenclature. *J. Proteomics* **235**, 104041 (2021).

3. M. Buckley, "Zooarchaeology by Mass Spectrometry (ZooMS) Collagen Fingerprinting for the Species Identification of Archaeological Bone Fragments" in *Zooarchaeology in Practice: Case Studies in Methodology and Interpretation in Archaeofaunal Analysis*, C. M. Giovas, M. J. LeFebvre, Eds. (Springer International Publishing, 2018), pp. 227–247.

4. M. Buckley, "Paleoproteomics: An Introduction to the Analysis of Ancient Proteins by Soft Ionisation Mass Spectrometry" in *Paleogenomics: Genome-Scale Analysis of Ancient DNA*, C. Lindqvist, O. P. Rajora, Eds. (Springer International Publishing, 2018), pp. 31–52.

5. F. Welker, Palaeoproteomics for human evolution studies. *Quat. Sci. Rev.* **190**, 137–147 (2018).

6. F. Welker, M. Soressi, W. Rendu, J.-J. Hublin, M. Collins, Using ZooMS to identify fragmentary bone from the Late Middle/Early Upper Palaeolithic sequence of Les Cottés, France. *Journal of Archaeological Science* **54**, 279–286 (2015).

7. M. Eda, M. Morimoto, T. Mizuta, T. Inoué, ZooMS for birds: Discrimination of Japanese archaeological chickens and indigenous pheasants using collagen peptide fingerprinting. *Journal of Archaeological Science: Reports* **34**, 102635 (2020).

8. M. Buckley, *et al.*, Species identification of archaeological marine mammals using collagen fingerprinting. *J. Archaeol. Sci.* **41**, 631–641 (2014).

9. V. Sinet-Mathiot, *et al.*, Combining ZooMS and zooarchaeology to study Late Pleistocene hominin behaviour at Fumane (Italy). *Sci. Rep.* **9**, 12350 (2019).

10. M. Buckley, *et al.*, Distinguishing between archaeological sheep and goat bones using a single collagen peptide. *J. Archaeol. Sci.* **37**, 13–20 (2010).

11. K. J. Gron, P. Rowley-Conwy, T. Z. T. Jensen, A. J. Taurozzi, A. Marciniak, Separating caprine (Capra / Ovis) distal tibiae: A case study from the Polish Neolithic. *Int. J. Osteoarchaeol.* **30**, 170–179 (2020).

12. S. E. Pilaar Birch, A. Scheu, M. Buckley, C. Çakırlar, Combined osteomorphological, isotopic, aDNA, and ZooMS analyses of sheep and goat remains from Neolithic Ulucak, Turkey. *Archaeol. Anthropol. Sci.* **11**, 1669–1681 (2019).

13. W. Taylor, *et al.*, Early pastoral economies along the Ancient Silk Road: Biomolecular evidence from the Alay Valley, Kyrgyzstan. *PLoS One* **13**, e0205646 (2018).

14. C. Culley, *et al.*, Iron Age hunting and herding in coastal eastern Africa: ZooMS identification of domesticates and wild bovids at Panga ya Saidi, Kenya. *J. Archaeol. Sci.* **130**, 105368 (2021).

15. A. N. Coutu, *et al.*, Palaeoproteomics confirm earliest domesticated sheep in southern Africa ca. 2000 BP. *Sci. Rep.* **11**, 6631 (2021).

16. E. Ananyevskaya, M. Buckley, M. Pal Chowdhury, K. Tabaldiev, G. Motuzaite Matuzeviciute, Specialized wool production economy of prehistoric farmstead of Chap I in the highlands of

Central Tian Shan (Kyrgyzstan). *Int. J. Osteoarchaeol.* **31**, 18–28 (2021).

17. C. Culley, *et al.*, Collagen fingerprinting traces the introduction of caprines to island Eastern Africa. *R Soc Open Sci* **8**, 202341 (2021).

18. S. Fiddyment, M. Collins, From field to frame. The contribution of bioarchaeological methods to understanding parchment production. *Gaz. du livre médiév.* **63**, 55–63 (2017).

19. S. Fiddyment, *et al.*, Animal origin of 13th-century uterine vellum revealed using noninvasive peptide fingerprinting. *Proc. Natl. Acad. Sci. U. S. A.* **112**, 15066–15071 (2015).

20. M. D. Teasdale, *et al.*, The York Gospels: a 1000-year biological palimpsest. *R Soc Open Sci* **4**, 170988 (2017).

21. S. P. Doherty, S. Henderson, S. Fiddyment, J. Finch, M. J. Collins, Scratching the surface: the use of sheepskin parchment to deter textual erasure in early modern legal deeds. *Heritage Science* **9**, 29 (2021).

22. A. Janzen, *et al.*, Distinguishing African bovids using Zooarchaeology by Mass Spectrometry (ZooMS): New peptide markers and insights into Iron Age economies in Zambia. *PLoS One* **16**, e0251061 (2021).

23. Y. van den Hurk, L. Spindler, K. McGrath, C. Speller, Medieval Whalers in the Netherlands and Flanders: Zooarchaeological Analysis of Medieval Cetacean Remains. *Environ. Archaeol.*, 1–15 (2020).

24. V. L. Harvey, *et al.*, Preserved collagen reveals species identity in archaeological marine turtle bones from Caribbean and Florida sites. *R Soc Open Sci* **6**, 191137 (2019).

25. D. Borić, *et al.*, Neanderthals on the Lower Danube: Middle Palaeolithic evidence in the Danube Gorges of the Balkans. *J. Quat. Sci.* (2021) https:/doi.org/10.1002/jqs.3354.

26. G. Pothier Bouchard, J. Riel-Salvatore, F. Negrino, M. Buckley, Archaeozoological, taphonomic and ZooMS insights into The Protoaurignacian faunal record from Riparo Bombrini. *Quat. Int.* **551**, 243–263 (2020).

27. M. E. Prendergast, E. M. Quintana Morales, A. Crowther, M. C. Horton, N. L. Boivin, Dietary Diversity on the Swahili Coast: The Fauna from Two Zanzibar Trading Locales. *Int J Osteoarchaeol* **27**, 621–637 (2017).

28. M. E. Prendergast, *et al.*, Reconstructing Asian faunal introductions to eastern Africa from multi-proxy biomolecular and archaeological datasets. *PLoS One* **12**, e0182565 (2017).

29. E. Guiry, M. Buckley, Urban rats have less variable, higher protein diets. *Proc. Biol. Sci.* **285** (2018).

30. T. Oueslati, *et al.*, 1st century BCE occurrence of chicken, house mouse and black rat in Morocco: Socio-economic changes around the reign of Juba II on the site of Rirha. *J. Archaeol. Sci. Rep.* **29**, 102162 (2020).

31. V. Slon, *et al.*, The genome of the offspring of a Neanderthal mother and a Denisovan father. *Nature* **561**, 113–116 (2018).

32. S. Brown, *et al.*, Identification of a new hominin bone from Denisova Cave, Siberia using collagen fingerprinting and mitochondrial DNA analysis. *Sci. Rep.* **6**, 23559 (2016).

33. S. Brown, *et al.*, Zooarchaeology through the lens of collagen fingerprinting at Denisova Cave.

Sci. Rep. **11**, 15457 (2021).

34. S. Brown, *et al.*, The earliest Denisovans and their cultural adaptation. *Nat Ecol Evol* (2021) https:/doi.org/10.1038/s41559-021-01581-2.

35. S. Charlton, *et al.*, Finding Britain's last hunter-gatherers: A new biomolecular approach to "unidentifiable" bone fragments utilising bone collagen. *Journal of Archaeological Science* **73**, 55–61 (2016).

36. F. Welker, *et al.*, Palaeoproteomic evidence identifies archaic hominins associated with the Châtelperronian at the Grotte du Renne. *Proc. Natl. Acad. Sci. U. S. A.* **113**, 11162–11167 (2016).

37. M. Buckley, V. L. Harvey, A. T. Chamberlain, Species identification and decay assessment of Late Pleistocene fragmentary vertebrate remains from Pin Hole Cave (Creswell Crags, UK) using collagen fingerprinting. *Boreas* **46**, 402–411 (2017).

38. K. McGrath, *et al.*, Identifying Archaeological Bone via Non-Destructive ZooMS and the Materiality of Symbolic Expression: Examples from Iroquoian Bone Points. *Sci. Rep.* **9**, 11027 (2019).

39. N. L. Martisius, *et al.*, Non-destructive ZooMS identification reveals strategic bone tool raw material selection by Neandertals. *Sci. Rep.* **10**, 7746 (2020).

40. J.-M. Pétillon, *et al.*, A Gray Whale in Magdalenian Perigord. Species identification of a bone projectile point from La Madeleine (Dordogne, France) using collagen fingerprinting. *Paléo*, 230–242 (2019).

41. A. Desmond, *et al.*, ZooMS identification of bone tools from the North African Later Stone Age. *J. Archaeol. Sci.* **98**, 149–157 (2018).

42. J. Bradfield, T. Forssman, L. Spindler, A. R. Antonites, Identifying the animal species used to manufacture bone arrowheads in South Africa. *Archaeol. Anthropol. Sci.* **11**, 2419–2434 (2019).

43. T. Z. T. Jensen, *et al.*, An integrated analysis of Maglemose bone points reframes the Early Mesolithic of Southern Scandinavia. *Sci. Rep.* **10**, 17244 (2020).

44. L. Moreau, *et al.*, Adaptive Trade-offs Towards the Last Glacial Maximum in North-Western Europe: a Multidisciplinary View from Walou Cave. *Journal of Paleolithic Archaeology* **4**, 11 (2021).

45. A. Tomasso, *et al.*, Gravettian weaponry: 23,500-year-old evidence of a composite barbed point from Les Prés de Laure (France). *J. Archaeol. Sci.* **100**, 158–175 (2018).

46. K. Adamczak, *et al.*, New evidence for deer valorisation by the TRB farmers from Poland using ZooMS and micro-CT scanning. *Journal of Archaeological Science: Reports* **40**, 103230 (2021).

47. T. Z. T. Jensen, *et al.*, The biomolecular characterization of a finger ring contextually dated to the emergence of the Early Neolithic from Syltholm, Denmark. *R Soc Open Sci* **7**, 191172 (2020).

48. I. C. C. von Holstein, *et al.*, Searching for Scandinavians in pre-Viking Scotland: molecular fingerprinting of Early Medieval combs. *Journal of Archaeological Science* **41**, 1–6 (2014).

49. S. P. Ashby, A. N. Coutu, S. M. Sindbæk, Urban Networks and Arctic Outlands: Craft Specialists and Reindeer Antler in Viking Towns. *European Journal of Archaeology* **18**, 679–704 (2015).

50. L. Ø. Brandt, U. Mannering, Taxonomic identification of Danish Viking Age shoes and skin objects by ZooMS (Zooarchaeology by mass spectrometry). *J. Proteomics* **231**, 104038 (2021).

51. J. A. Ebsen, K. Haase, R. Larsen, D. V. P. Sommer, L. Ø. Brandt, Identifying archaeological leather – discussing the potential of grain pattern analysis and zooarchaeology by mass spectrometry (ZooMS) through a case study involving medieval shoe parts from Denmark. *Journal of Cultural Heritage* **39**, 21–31 (2019).

52. N. Ruffini-Ronzani, *et al.*, A biocodicological analysis of the medieval library and archive from Orval Abbey, Belgium. *R Soc Open Sci* **8**, 210210 (2021).

53. J. Vnouček, *et al.*, "The parchment of the Vienna Genesis: characteristics and manufacture" in *The Vienna Genesis Material Analysis and Conservation of a Late Antique Illuminated Manuscript on Purple Parchment*, (Boehlau Verlag GmbH & Co. KG, 2020), pp. 35–70.

54. C. D. Calvano, E. C. L. Rigante, T. R. I. Cataldi, L. Sabbatini, In Situ Hydrogel Extraction with Dual-Enzyme Digestion of Proteinaceous Binders: the Key for Reliable Mass Spectrometry Investigations of Artworks. *Anal. Chem.* **92**, 10257–10261 (2020).

55. C. D. Calvano, E. Rigante, R. A. Picca, T. R. I. Cataldi, L. Sabbatini, An easily transferable protocol for in-situ quasi-non-invasive analysis of protein binders in works of art. *Talanta* **215**, 120882 (2020).

56. T. Tripković, *et al.*, Identification of protein binders in artworks by MALDI-TOF/TOF tandem mass spectrometry. *Talanta* **113**, 49–61 (2013).

57. R. Hynek, S. Kuckova, J. Hradilova, M. Kodicek, Matrix-assisted laser desorption/ionization time-of-flight mass spectrometry as a tool for fast identification of protein binders in color layers of paintings. *Rapid Commun. Mass Spectrom.* **18**, 1896–1900 (2004).

58. S. Kuckova, R. Hynek, M. Kodicek, Identification of proteinaceous binders used in artworks by MALDI-TOF mass spectrometry. *Anal. Bioanal. Chem.* **388**, 201–206 (2007).

59. D. P. Kirby, M. Buckley, E. Promise, S. A. Trauger, T. R. Holdcraft, Identification of collagen-based materials in cultural heritage. *Analyst* **138**, 4849–4858 (2013).

60. S. Fiddyment, *et al.*, Girding the loins? Direct evidence of the use of a medieval English parchment birthing girdle from biomolecular analysis. *R Soc Open Sci* **8**, 202055 (2021).

61. D. P. Kirby, A. Manick, R. Newman, Minimally Invasive Sampling of Surface Coatings for Protein Identification by Peptide Mass Fingerprinting: A Case Study with Photographs. *J. Amer. Inst. Conserv.* **59**, 235–245 (2020).

62. J. Ma, *et al.*, The Mammuthus-Coelodonta Faunal Complex at its southeastern limit: A biogeochemical paleoecology investigation in Northeast Asia. *Quat. Int.* **591**, 93–106 (2021).

63. N. Amano, Y. V. Wang, N. Boivin, P. Roberts, "Emptying forests?" conservation implications of past human-primate interactions. *Trends Ecol. Evol.* **36**, 345–359 (2021).

64. V. L. Harvey, *et al.*, Interpreting the historical terrestrial vertebrate biodiversity of Cayman Brac (Greater Antilles, Caribbean) through collagen fingerprinting. *The Holocene* **29**, 531–542 (2019).

65. K. K. Richter, *et al.*, What's the catch? Archaeological application of rapid collagen-based species identification for Pacific Salmon. *Journal of Archaeological Science* **116**, 105116 (2020).

66. T. Rick, V. L. Harvey, M. Buckley, Collagen fingerprinting and the Chumash billfish fishery, Santa Barbara Channel, California, USA. *Archaeol. Anthropol. Sci.* **11**, 6639–6648 (2019).

67. R. M. Winter, W. de Kock, P. J. Palsbøll, C. Çakirlar, Potential applications of biomolecular archaeology to the ecohistory of sea turtles and groupers in Levant coastal antiquity. *Journal of Archaeological Science: Reports* **36**, 102872 (2021).

68. E. G. Garrison, G. S. Morgan, K. McGrath, C. Speller, A. Cherkinsky, Recent dating of extinct Atlantic gray whale fossils, (Eschrichtius robustus), Georgia Bight and Florida, western Atlantic Ocean. *PeerJ* **7**, e6381 (2019).

69. L. G. van der Sluis, *et al.*, Combining histology, stable isotope analysis and ZooMS collagen fingerprinting to investigate the taphonomic history and dietary behaviour of extinct giant tortoises from the Mare aux Songes deposit on Mauritius. *Palaeogeogr. Palaeoclimatol. Palaeoecol.* **416**, 80–91 (2014).

70. V. L. Harvey, L. Daugnora, M. Buckley, Species identification of ancient Lithuanian fish remains using collagen fingerprinting. *Journal of Archaeological Science* **98**, 102–111 (2018).

71. A. N. Coutu, G. Whitelaw, P. le Roux, J. Sealy, Earliest Evidence for the Ivory Trade in Southern Africa: Isotopic and ZooMS Analysis of Seventh–Tenth Century and Ivory from KwaZulu-Natal. *African Archaeological Review* **33**, 411–435 (2016).
